# Observation of the clinical characteristics of chronic diarrhoea in children and evaluation of the prognostic value of nutritional and immune indicators

**DOI:** 10.5937/jomb0-48174

**Published:** 2024-11-16

**Authors:** Jianyun Hao, Xin Ma, Youzhe Gong, Dan Zhu, Huijuan Ning, Xuemei Zhong

**Affiliations:** 1 Children's Hospital of Capital Institute of Pediatrics, Department of Gastroenterology, Beijing City, China

**Keywords:** chronic diarrhoea, clinical features, nutritional status, immune indicators, prognosis, diagnostic efficacy, hronična dijareja, kliničke karakteristike, status uhranjenosti, imunološki indikatori, prognoza, dijagnostička efikasnost

## Abstract

**Background:**

This research aimed to assess the clinical characteristics of chronic diarrhoea in children and explore the prognostic value of nutritional status and immune indicators.

**Methods:**

A total of 190 patients with chronic diarrhoea from January 2017 to June 2020 were enrolled to analyze their epidemiology. The patients were divided into a better prognosis group (cured and improved) and a poor prognosis group (uncured). The efficacy of nutritional status and immune indicators in children's chronic diarrhoea prognosis was analyzed.

**Results:**

Most patients were 0-3 years old (74.2%), of which 54.3% were less than 1 year old, and 48.95% had a course of 1 to 2 months. The albumin, immunoglobulin G (IgG), IgA and IgM levels, albumin, globulin, and the ratio of albumin to globulin in the better prognosis group were higher than those in the poor prognosis group. The AUC (area under the curve) of the combined detection in evaluating the prognosis of children with chronic diarrhoea was greater than that of the albumin and globulin alone (P<0.05). IgG 10.05 g/L and IgA 7.72 g/L were protective factors affecting the prognosis of children with chronic diarrhoea.

**Conclusions:**

Children with chronic diarrhoea are mainly infants and young children with various clinical symptoms and are prone to comorbidities such as malnutrition, anemia, hypoalbuminemia, and impaired immune function. In evaluating the prognosis of children, evaluating nutritional status and immune indicators together is valuable.

## Abstract (sr)

**Uvod:**

Ovo istraživanje je imalo za cilj da proceni kliničke karakteristike hronične dijareje kod dece i istraži prognostičku vrednost nutritivnog statusa i imunoloških indikatora.

**Metode:**

Ukupno 190 pacijenata sa hroničnom dijarejom je bilo uključeno u analizu njihove epidemiologije od januara 2017. do juna 2020. Pacijenti su podeljeni u grupu sa boljom prognozom (izlečeni pacijenti i poboljšano stanje) i grupu sa lošom prognozom (neizlečeni). Analizirana je efikasnost nutritivnog statusa i imunoloških indikatora u prognozi hronične dijareje kod dece.

**Rezultati:**

Većina pacijenata je imala 0-3 godine (74,2%), od kojih je 54,3% bilo mlađe od 1 godine, a 48,95% bilo između starosti od 1 do 2 meseca. Nivoi albumina, imunoglobulina G (IgG), IgA i IgM, albumina, globulina i odnos albumina prema globulinu u grupi sa boljom prognozom bili su viši od onih u grupi sa lošom prognozom. AUC (površina ispod krive) kombinovane detekcije u proceni prognoze bolesti kod dece sa hroničnom dijarejom je bila veća od one koje se tiče samo albumina i globulina (P<0,05). IgG 10,05 g/L i IgA 7,72 g/L su zaštitni faktori koji utiču na prognozu bolesti kod dece sa hroničnom dijarejom.

**Zaključak:**

Deca sa hroničnom dijarejom su uglavnom odojčad i mala deca sa različitim kliničkim simptomima i sklona su pratećim bolestima kao što su pothranjenost, anemija, hipoalbuminemija i oštećena imunološka funkcija. U proceni prognoze bolesti kod te dece, bitna je zajednička procena nutritivnog statusa i imunoloških indikatora.

## Introduction

As a frequently-occurring disease in paediatrics, diarrhoea is the cause of high mortality, accounting for 2 billion diarrhoea cases according to the statistics of the United Nations Children’s Fund (UNICEF) and the World Health Organization (WHO) [1]. Caused by various pathogens and factors, diarrhoea is a group of diseases characterized by increased stool frequency and changes in stool characteristics and is the main cause of illness and death in children [2] [3]. It is believed that demographic characteristics in different regions contribute to the difference in the etiology of chronic diarrhoea in children, which can ultimately affect the formulation of diagnosis and treatment plans. Therefore, analyzing the epidemiology of pediatric diarrhoea was crucial for clinical prevention and treatment [4]. In clinical practice, chronic diarrhoea is more common in children with various manifestations and complex etiologies and often leads to malnutrition and immune dysfunction, seriously threatening the life, health, and safety of children. Relevant reports also have pointed out that children with chronic diarrhoea may develop malnutrition due to intestinal mucosal damage and nutrient absorption disorders [5] [6]. Malnutrition can lead to anemia, hypoalbuminemia, and low immune function. However, the relationship between nutritional status, immune function, and prognosis of children is still in the exploratory stage. In this study, a retrospective study was conducted on the clinical data of children with chronic diarrhoea admitted to our hospital in the past 4 years, and the incidence, immune function, and nutritional status were assessed to provide some ideas for clinical diagnosis and treatment.

## Materials and methods

### Clinical data

A total of 190 patients with chronic diarrhoea who were hospitalized in the Department of Gastroenterology, Children’s Hospital Affiliated with the Capital Institute of Pediatrics from January 2017 to June 2020 were enrolled. Inclusion criteria: 1) Meeting the diagnostic criteria (abnormal increase in stool moisture and stool frequency, usually more than 3 times within 24 h; stool condition is more important than stool frequency; Chronic diarrhoea refers to the duration of diarrhoea for 14 days or more) [7] [8]. 2) Complete clinical data; 3) 0 months to 17 years old. Exclusion criteria: 1) hospitalization time 24 h; 2) incomplete clinical data; 3) immune diseases; 4) malnutrition caused by other diseases. All guardians were well-informed of the collection of clinical samples and signed the informed consent form before the study. The Ethics Committee approved this study in our hospital.

### Methods

### Clinical data collection

The hospital information system collected age, course of disease, and disease characteristics.

### Nutritional status and indicators

The children’s height (length) and weight were measured within 24 hours of admission. The weight is accurate to 0.1 kg, and the length/height is accurate to 0.1 cm. The nutritional status of children aged 5 years (60 months) and younger was evaluated according to the weight-for-age Z score published by WHO in 2006 [9], while that of children aged 5–18 years old was evaluated according to Z-score (10). Z-score <-1SD for any three items = mild malnutrition; <-2SD = moderate malnutrition; <-3SD = severe malnutrition.

The albumin level was measured by the bromocresol green method (Genesource, Shanghai, China) while the serum globulin (intra-assay CV: 7.8%, inter-assay CV: 11.3%, Sensitivity: 8.624 pg/mL, range: 18.75 pg/mL - 1200 pg/mL) was tested by the enzyme-linked immunosorbent assay (ELISA) kit (Jingmei Biotechnology, Jiangsu, China).

### Immune indicators

The serum immunoglobulin level of patients was detected by ELISA kit (Jingmei Biotechnology, Jiangsu, China), including immunoglobulin G (IgG, intra-assay CV: 6.4%, inter-assay CV: 14.7%, Sensitivity: 0.02 ng/mL, range: 0.23 ng/mL - 15 ng/mL), IgA (intra-assay CV: 4.12%, inter-assay CV:5.92%, Sensitivity: 0.25 ng/mL, range: 0.78 ng/mL - 50 ng/mL) and IgM (intra-assay CV: 4.9%, inter-assay CV: 5.8%, Sensitivity: 50 pg/mL, range: 390.63 pg/mL - 25000 pg/mL).

### Prognostic evaluation

Patients were given montmorillonite powder (Manufacturer: Yangtze River Pharmaceutical (Group) Co., Ltd.; H20053263; Specification: 3 g of montmorillonite per bag; Production batch No.: 11041312; Dosage: 1-year-old children with chronic diarrhoea, 3 g /times, 1 bag/d; 1–2 years old children with chronic diarrhoea, 3 g/times, 1–2 bags/d; 2 years old children with chronic diarrhoea, 3 g/time, 2–3 bags/d) in 3 doses. The criteria for evaluating the efficacy of chronic diarrhoea in children are as follows [10]: cured = symptoms, frequency of defecation, and the amount and condition of stool become normal; improved = clinical symptoms are significantly improved, and the frequency of defecation is reduced to 1–3 times/d, the stool condition is improved; uncured = patients do not meet the above standards. The patients were divided into a better prognosis group (cured and improved) and a poor prognosis group (uncured).

### Observation indicators

(1) the epidemiological characteristics of children with chronic diarrhoea. (2) the serum nutritional and immune function indexes and the prognostic value of the indices.

### Statistical analysis

Data were registered using an Excel form, and SPSS 25.0 statistical software (IBM Corp., Armonk, NY, USA) was used for data analysis. X2 and t-tests compared the enumeration and measurement data; the diagnostic efficacy was analyzed by the receiver operating characteristics (ROC) curve, and the risk factors were assessed by logistic regression test. *P*<0.05 indicated that the difference was statistically significant.

## Results

### Age distribution of chronic diarrhoea

**Figure 1:**
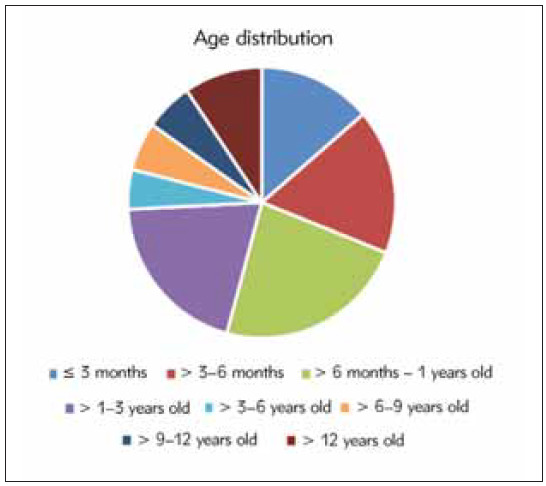
The age distribution of the children with chronic diarrhoea.

There were 118 males and 72 females with a median age of 11.2 months ranging from 1 month to 17 years. The age distribution of the children is shown in Table 1 and Figure 1.

**Table 1:** Age distribution of chronic diarrhoea in children.

Distribution	Cases	Percentage (%)
≤3 months	26	13.68
3–6 months	33	17.37
6 months–1 years	44	23.16
1–3 years	38	20
3–6 years	9	4.74
6–9 years	11	5.79
9–12 years	11	5.79
>12 years	18	9.47

### Distribution of diarrhoea occurrence

**Figure 2:**
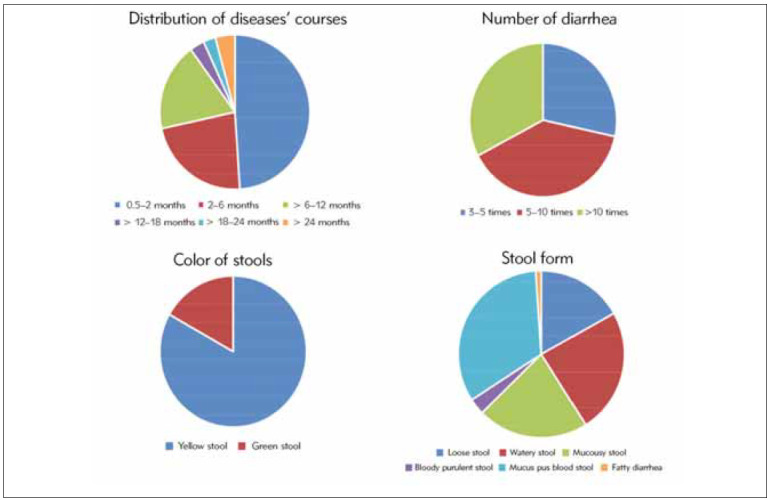
The distribution of diarrhoea occurrence in children with chronic diarrhoea.

The duration of diarrhoea was 0.5 to 144 months (12 years), with a median of 2.94 months. The course of diarrhoea, the frequency of diarrhoea, stool colour, and stool condition are shown in Table 2 and Figure 2.

**Table 2:** Distribution of diarrhoea occurrence in patients with chronic diarrhoea.

Distribution	Cases	Percentage (%)
Course of disease		
1–2 months	93	48.95
2–6 months	43	22.63
6–12 months	35	18.42
12–18 months	6	3.16
18–24 months	5	2.63
>24 months	8	4.21
Frequency of diarrhoea		
3–5	54	28.42
5–10	74	38.95
>10	62	32.63
Stool colour		
Yellow	158	83.16
Green	32	16.84
Stool character		
Loose stool	32	16.84
Watery stool	46	24.21
Mucous stool	41	21.58
Bloody purulent stool	6	3.16
Mucous and bloody purulent stool	63	33.16
Steatorrhea	2	1.05

### Clinical symptoms and accompanying diseases

**Figure 3:**
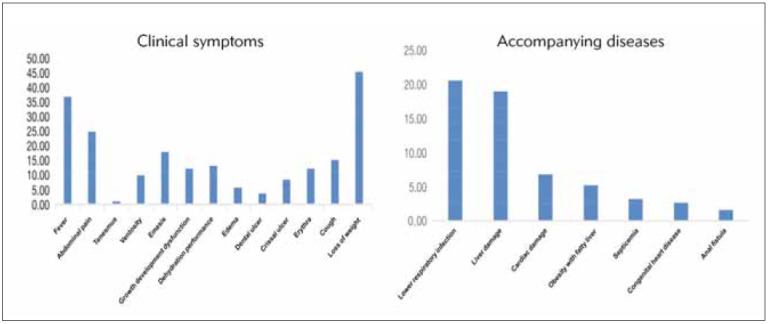
The clinical symptoms and accompanying diseases in children with chronic diarrhoea.

The clinical symptoms and accompanying diseases in children with chronic diarrhoea are shown inTable 3 and Figure 3. Among the 190 patients with chronic diarrhoea, concomitant symptoms included fever in 70 cases, stomachache in 47 cases, tenesmus in 2 cases, bloating in 19 cases, vomiting in 34 cases, growth disorder in 23 cases, dehydration in 25 cases, edema in 11 cases, mouth ulcers in 7 cases, perianal ulcer in 16 cases, rash in 23 cases, cough in 29 cases, weight loss in 86 cases. In addition, 39 children with chronic diarrhoea were combined with lower respiratory tract infection, 46 children with chronic diarrhoea were combined with liver damage, 13 children with chronic diarrhoea were combined with myocardial damage, 6 children with chronic diarrhoea were combined with septicemia, 5 children with chronic diarrhoea were combined with congenital heart disease, and 3 children with chronic diarrhoea were combined with anal fistula.

**Table 3:** Clinical symptoms and accompanying diseases in patients with chronic diarrhoea.

Distribution	Cases	Percentage (%)
Clinical symptoms		
Fever	70	36.84
Stomachache	47	24.74
Tenesmus	2	1.05
Bloating	19	10
Vomiting	34	17.89
Growth disorder	23	12.11
Dehydration	25	13.16
Edema	11	5.79
Mouth ulcers	7	3.68
Perianal ulcer	16	8.42
Rash	23	12.11
Cough	29	15.26
Weight loss	86	45.26
Accompanying diseases		
Lower respiratory tract infection	39	20.53
Liver damage	46	24.21
Myocardial damage	13	6.84
Septicemia	6	3.16
Congenital heart disease	5	2.63
Anal fistula	3	1.58

### Stool examination and immune function examination

All children underwent at least 2 stool cultures, and the results of the fecal examination are shown in Table 4. Specifically, 29 strains of pathogenic bacteria were detected, including Shigella in 8 cases, Salmonella in 6 cases, pseudomonas aeruginosa in 1 case, Escherichia coli O157 in 1 case, Candida Albicans in 6 cases, Candida tropical in 5 cases, and Candida glabrata in 2 cases. Fecal smear-modified antacid staining microscopy was performed in 8 cases, and cryptosporidiosis was observed in 1 case. In addition, immune function tests were also performed. Specifically, 104 cases were tested for immunoglobulin and antibody, and 61 cases were abnormal. Lymphocyte subpopulation test was performed in 73 cases, and 42 cases were abnormal. Leukocyte phagocytosis test was performed in 65 cases, and there was no abnormality.

**Table 4:** Stool test results.

Infectious factors	Cases	Percentage (%)
Bacterial infection	16	8.42
Shigella	8	
Salmonella	6	
Pseudomonas aeruginosa	1	
Escherichia coli O157	1	
Fungal infection	13	6.84
Candida Albicans	6	
Candida tropical	5	
Candida glabrata	2	
Protozoan infection	1	0.53
Cryptosporidiosis	1	

### Etiology analysis

The causes of chronic diarrhoea include food protein-induced allergic proctocolitis, infectious diarrhoea (bacterial, fungal, or protozoal infections), food protein-induced enteropathy, ulcerative colitis, Crohn’s disease, very early-onset inflammatory bowel disease, short- bowel syndrome, irritable bowel syndrome, primary enteric lymphangiectasis, secondary infection of colon polyps, antibiotic-associated diarrhoea, functional diarrhoea, congenital tufting enteropathy, diacylglycerol transferase 1 deficiency, eosinophilic gastroenteritis, X-linked lymphoproliferative disease, progressive familial intrahepatic cholestasis, acrodermatitis enteropathica, celiac disease, and unknown cause (Table 5).

**Table 5:** Etiology analysis.

Etiology	Cases	Percentage (%)
Food protein-induced allergic proctocolitis	49	25.79
Infectious diarrhoea (bacterial, fungal, or protozoal infections)	30	15.79
Food protein-induced enteropathy	13	6.84
Ulcerative colitis	12	6.32
Crohn’s disease	12	6.32
Very early-onset inflammatory bowel disease	10	5.26
Short-bowel syndrome	7	3.68
Irritable bowel syndrome	7	3.68
Primary enteric lymphangiectasis	6	3.16
Secondary infection of colon polyps	5	2.63
Antibiotic-associated diarrhoea	4	2.11
Functional diarrhoea	4	2.11
Congenital tufting enteropathy	3	1.58
Diacylglycerol transferase 1 deficiency	3	1.58
Eosinophilic gastroenteritis	2	1.05
X-linked lymphoproliferative disease	1	0.53
Progressive familial intrahepatic cholestasis	1	0.53
Acrodermatitis enteropathica	1	0.53
Celiac disease	1	0.53
Unknown causes	19	10.00

### Nutritional status

**Figure 4:**
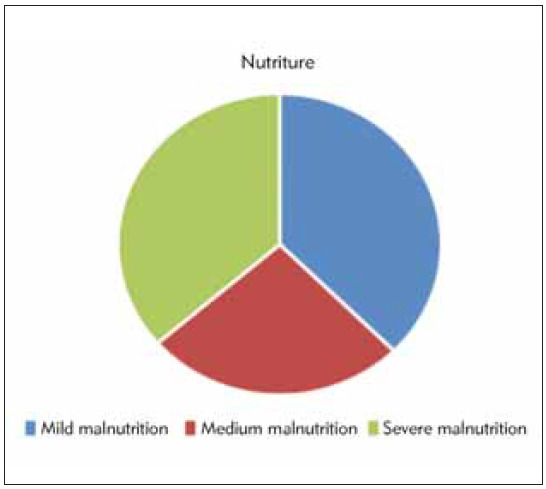
The nutritional status of the children with chronic diarrhoea.

As demonstrated in Figure 4, there were 88 malnourished patients, accounting for 46.3% (88/190), among which mild malnutrition accounted for 37.5% (33 cases), moderate malnutrition accounted for 26.1% (23 cases), and severe malnutrition accounted for 36.4% (32 cases). In addition, 87 cases of anemia were found, including 7 cases with Hb of 30–60 g/L, 44 cases with Hb of >60–90 g/L, and 36 cases with Hb of >90–120 g/L; 69 cases of hypoproteinemia; 25 cases of electrolyte disorders; and 14 cases of metabolic acidosis.

### The nutritional status of the children with chronic diarrhoea in the better prognosis group and poor prognosis group

**Figure 5:**
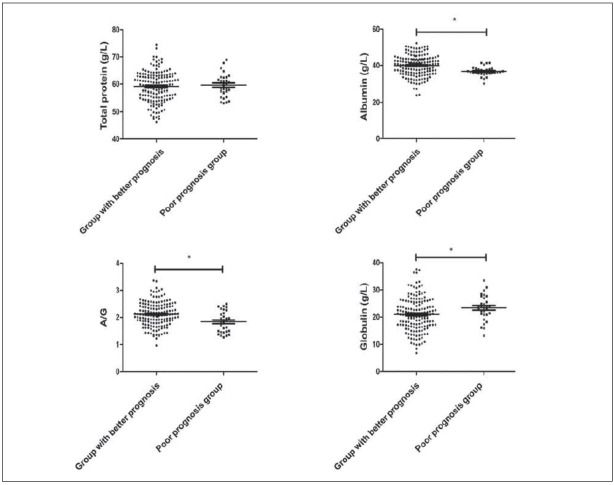
The nutritional status of the children with chronic diarrhoea in the better prognosis group and poor prognosis group, * P<0.05.

The patients were divided into a good prognosis group (21 cases cured, 138 cases improved, a total of 159 cases) and a poor prognosis group (30 cases uncured, 1 case death, a total of 31 cases). The albumin and ratio of albumin to globulin in the better prognosis group were higher than those in the poor prognosis group, but globulin level was lower than that in the poor prognosis group (*P*<0.05); there was no significant difference in total protein levels between the two groups (*P*>0.05), as shown in Figure 5.

### Immune indexes in the children with chronic diarrhoea in the better prognosis group and poor prognosis group

**Figure 6:**
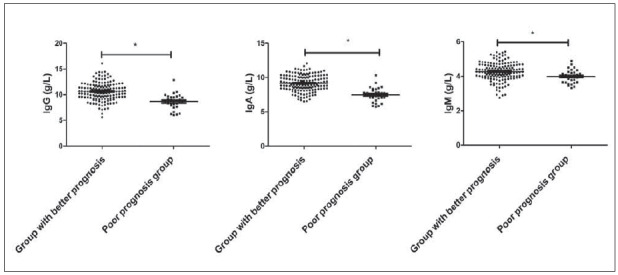
Immune indexes in the children with chronic diarrhoea in the better prognosis group and poor prognosis group, * P<0.05.

The levels of IgG, IgA, and IgM in the good prognosis group were higher than those in the poor prognosis group (*P*<0.05, Figure 6).

### Diagnostic efficacy of nutritional status and immune indicators in the prognosis of children with chronic diarrhoea

**Figure 7:**
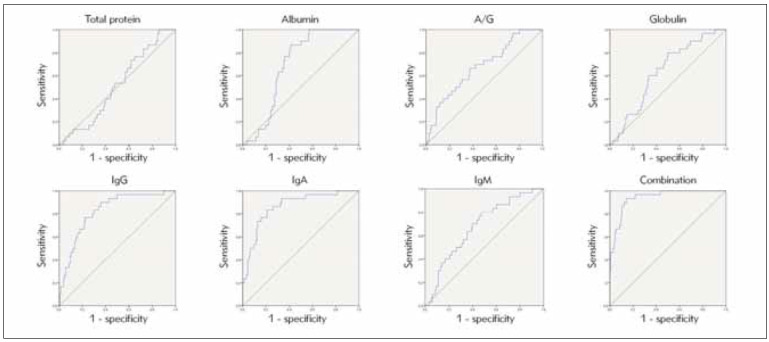
Diagnostic efficacy of nutritional status and immune indicators in the prognosis of children with chronic diarrhoea using the ROC method.

According to the ROC method, the total protein, albumin, ratio of albumin to globulin, globulin, IgG, IgA, and IgM had a good efficacy in the prognosis of children with chronic diarrhoea (all *P*<0.05). The AUC of the combined values was greater than the indicators, including the total protein, albumin, ratio of albumin to globulin, globulin, and IgM, with the AUC of 0.941 (95%CI: 0.903–0.979), as shown in Table 6 and Figure 7.

**Table 6:** Prognostic value of nutritional status and immune indicators. Note: * P<0.05 compared to combined.

Indicators	Cutoff value	AUC	SE	95%CI
Total protein	59.58 g/L	0.512*	0.051	0.412–0.612
Albumin	38.91 g/L	0.693*	0.038	0.618–0.768
Ratio of albumin to globulin	2.04 g/L	0.678*	0.052	0.576–0.780
Globulin	20.97 g/L	0.634*	0.049	0.537–0.731
IgG	10.05 g/L	0.823	0.039	0.746–0.899
IgA	7.72 g/L	0.863	0.036	0.793–0.933
IgM	4.09 g/L	0.677*	0.05	0.580–0.775
Combined		0.941	0.019	0.903–0.979

### Logistic regression analysis of the nutritional status and immune indexes and prognosis of children with chronic diarrhoea

Ig 10.05 g/L (OR: 0.552; 95% CI: 0.432–0.705; P<0.001) and IgA 7.72 g/L (OR: 0.508; 95% CI: 0.339–0.762; P=0.001) were protective factors affecting the prognosis of children with chronic diarrhoea, see Table 7.

**Table 7:** Logistic regression analysis of the nutritional status and immune indexes and prognosis of children with chronic diarrhea. Assignment: total protein (59.58g/L is 1, <59.58 g/L is 0); albumin (38.91 g/L is 1, <38.91 g/L is 0); Ratio of albumin to globulin (2.04 g/L) 1, <2.04 g/L is 0); globulin (20.97 g/L is 1, <20.97 g/L is 0); IgG (10.05 g/L is 1, <10.05 g/L is 0); IgA (7.72g/L is 1, <7.72 g/L is 0); IgM (4.09 g/L is 1, <4.09 g/L is 0).

Indicators	β	SE	wald x^2^	OR	95%CI	P
Total protein	0.529	0.275	3.7	1.697	0.990–2.910	0.055
Albumin	-0.612	0.393	2.425	0.542	0.251–1.171	0.12
Ratio of albumin to globulin	-0.801	0.475	2.844	0.449	0.177–1.139	0.092
Globulin	0.587	0.309	3.609	1.799	0.982–3.296	0.058
IgG	-0.594	0.125	22.582	0.552	0.432–0.705	<0.001
IgA	-0.677	0.207	10.696	0.508	0.339-0.762	0.001
IgM	-0.493	0.312	2.497	0.611	0.331-1.126	0.115

## Discussion

It remains the third most common cause of death among children under five years old despite a decline in mortality since 1980 [11]. Chronic diarrhoea is becoming a hot spot of clinical attention due to its complex etiology and pathogenesis and difficult diagnosis and treatment. Chronic diarrhoea can cause growth retardation, malnutrition and cognitive impairment [12]. Therefore, effective treatments are of great significance in improving the quality of life and reducing the mortality rate [13] [14]. Children with chronic diarrhoea due to allergy, immunity, infection, lactose intolerance, intestinal lymphangiectasia and primary immunodeficiency disease have been mostly concentrated in patients under 3 years old [15] [16]. In this study, 54.3% of patients with chronic diarrhoea were under 1 year old, and 74.3% were under 3 years old, indicating that infants and young children were the main populations of chronic diarrhoea in children, which was consistent with relevant reports [17] [18]. Children with chronic diarrhoea are prone to weight loss, mainly related to abnormal intestinal mucosal function.

In recent years, although the incidence of diarrhoea in children in developing countries has decreased, nearly 3% to 20% of acute diarrhoea developed into chronic diarrhoea, especially in malnourished children, 20% of acute diarrhoea would develop into chronic diarrhoea. A relevant report has also pointed out that patients with malnutrition diarrhoea are prone to develop chronic diarrhoea [19]. Chronic diarrhoea often causes nutrient absorption disorders due to prolonged diarrhoea, which in turn leads to malnutrition, anemia, low immune function, even hypoproteinemia, and growth and development disorders. Malnutrition, anemia, and low immune function are easy to cause diarrhoea to persist and cause each other, resulting in a vicious circle [20] [21]. In this study, among the 190 patients with chronic diarrhoea, 88 cases were combined with malnutrition, and most of them showed moderate to severe malnutrition; 87 cases were combined with anemia, and most of them showed moderate anemia; 61 cases were immunoglobulin abnormality; 69 cases were hypoproteinemia, and 23 cases were growing development disorders. Thus, it can be seen that malnutrition, anemia, immunocompromise and hypoproteinemia are important factors for prolonging diarrhoea.

The protein level is an important indicator for evaluating the nutritional status of children [12]. In this study, there was no significant difference in the total protein level between the poor prognosis group and the better prognosis group, indicating that the total protein level has little significance for the evaluation of prognosis, which is inconsistent with a former study [22] possibly because of the small number of cases included in this study. Decreased serum albumin concentration is a characteristic change of malnutrition, but due to the long half-life of albumin, mild to moderate malnutrition changes little, and the sensitivity is not high. This study found that the albumin and globulin levels of the patients in the poor prognosis group decreased, which further indicated that the nutritional status of the poor prognosis patients was poor, suggesting that the abnormal nutritional status of the children should be detected early through laboratory tests and corrected in time.

The gastrointestinal tract of children is immature, and enterobacteria are susceptible to external factors and secrete many electrolytes, resulting in abdominal distension, abdominal pain and even diarrhoea [23]. Chronic diarrhoea can reduce the protective barrier formed by anaerobic bacteria in the body, causing a large number of pathogenic bacteria to grow and multiply, and gradually lead to the imbalance of intestinal flora, which can increase the water and electrolyte imbalance, and eventually lead to abnormal immune function [24]. Chronic diarrhoea can lead to immune dysfunction [25]. This study showed that the levels of IgG, IgA, and IgM in the better prognosis group were higher than those in the poor prognosis group, indicating that patients with poor prognosis had low immune function. In addition, this study found that IgG 10.05 g/L and IgA 7.72 g/L are protective factors affecting the prognosis of children with chronic diarrhoea, further indicating that the abnormal immune function of patients is an important factor leading to poor prognosis, which is mainly related to the decreased immune function affecting the imbalance of flora.

A variety of etiologies causes chronic diarrhoea in children. Finding and managing the etiology is of great significance for improving the cure rate and prognosis [26]. Relevant reports point out that malnutrition, anemia, decreased serum protein, and low immune function are responsible for persistent diarrhoea, and timely and reasonable correction of the above factors can promote the recovery of children with diarrhoea [27]. It is suggested that the prognosis may be predicted by detecting patients’ nutritional status and immune function. The results of this study showed that the AUC of the combined detection of each index to evaluate the prognosis of children with chronic diarrhoea was greater than that of albumin, globulin and other indicators alone, and the AUC was greater than 0.9, suggesting that the combined detection has predictive value for the prognosis of children.

To sum up, children with chronic diarrhoea are mainly infants and young children with various clinical symptoms and are prone to comorbidities such as malnutrition, anemia, hypoalbuminemia, and impaired immune function. The combined detection of nutritional status and immune indicators in children is of value in evaluating the prognosis of children.

## Dodatak

### List of abbreviations

IgG, immunoglobulin G; 

AUC, area under the curve; 

ELISA, enzyme-linked immunosorbent assay; 

ROC, receiver operating characteristics; 

UNICEF, United Nations Children’s Fund; 

WHO, World Health Organization, 

BMI, body mass index.

### Acknowledgements

Not applicable.

### Funding

Not applicable.

### Ethics approval and consent to participate

The present study was approved by the Ethics Committee of Children’s Hospital of Capital Institute of Pediatrics, and written informed consent was obtained from all patients before the start of the study. All procedures were performed following the ethical standards of the Institutional Review Board and The Declaration of Helsinki, and its later amendments or comparable ethical standards.

### Availability of data and materials

The data and materials used to support the findings of this study are available from the corresponding author.

### Authors’ contributions

JianYun designed the research study. Xin Ma performed the research. YouZhe Gong and Dan Zhu provided help and advice on the experiments. HuiJuan Ning analyzed the data. JianYun Hao and XueMei Zhong wrote the manuscript. All authors contributed to editorial changes in the manuscript. All authors read and approved the final manuscript.

### Conflict of interest statement

All the authors declare that they have no conflict of interest in this work.
